# Functional roles of conserved lncRNAs and circRNAs in eukaryotes

**DOI:** 10.1016/j.ncrna.2024.06.014

**Published:** 2024-06-25

**Authors:** Jingxin Li, Xiaolin Wang

**Affiliations:** Department of Clinical Laboratory, The First Affiliated Hospital of USTC, The RNA Institute, School of Basic Medical Sciences, Division of Life Science and Medicine, University of Science and Technology of China (UTSC), Hefei, 230027, Anhui, China

**Keywords:** lncRNA, circRNA, Conservation, Function, Mechanism

## Abstract

Long non-coding RNAs (lncRNAs) and circular RNAs (circRNAs) have emerged as critical regulators in essentially all biological processes across eukaryotes. They exert their functions through chromatin remodeling, transcriptional regulation, interacting with RNA-binding proteins (RBPs), serving as microRNA sponges, etc. Although non-coding RNAs are typically more species-specific than coding RNAs, a number of well-characterized lncRNA (such as *XIST* and *NEAT1*) and circRNA (such as *CDR1as* and *ciRS-7*) are evolutionarily conserved. The studies on conserved lncRNA and circRNAs across multiple species could facilitate a comprehensive understanding of their roles and mechanisms, thereby overcoming the limitations of single-species studies. In this review, we provide an overview of conserved lncRNAs and circRNAs, and summarize their conserved roles and mechanisms.

## 1 Introduction

A large portion of the genome is transcribed into non-coding RNA (ncRNA) molecules, which lack the potential or capacity to encode proteins [1,2]. Some ncRNAs shorter than 200 nucleotides (nts), such as transfer RNA (tRNA), tRNA-derived small RNA (tsRNA), small nucleolar RNA (snoRNA), enhancer RNA (eRNA), small nuclear ribonucleic acid RNA (snRNA), piwi-interacting RNA (piRNA), and microRNA (miRNA) have been reviewed elsewhere [[3], [4], [5], [6], [7]]. Long ncRNAs (lncRNAs), which are more than 200-nt in length, always fold into distinct secondary or tertiary structures to demonstrate their functional roles [[8], [9], [10]]. LncRNAs, often with 5’ caps and poly(A) tails, are a common class of endogenous RNA molecules generally transcribed by RNA polymerase II (Pol II) or other RNA polymerases [[9], [10], [11], [12]]. LncRNAs have an extensive expression pattern in a wide variety of species, including animals, plants, yeast, prokaryotes, and even viruses [13]. Compared to messenger RNAs (mRNAs), most lncRNAs demonstrate higher cell- and tissue-specificity but less conserved features among species [8,11,14]. Some lncRNAs reside in the nucleus to regulate gene expression *in cis*, and several lncRNAs localize to the cytoplasm to play roles *in trans* [12,15]. LncRNAs have been revealed as essential regulators of a multiplicity of biological processes, including transcriptional regulation, chromatin remodeling, mRNA stability regulation, mRNA exportation, interacting with RNA-binding proteins (RBPs), and competing endogenous RNAs (ceRNAs) [8,11,16,17].

Circular RNAs (circRNAs) are covalently closed single-stranded RNA molecules that are the output of transcription and RNA processing in eukaryotes [2,11,[17], [18], [19], [20], [21]]. They are backspliced from pre-RNAs, which are facilitated by reverse complementary sequence and RBPs, or other RNA circularization methods [19,20,[22], [23], [24], [25], [26]]. CircRNAs are primarily localized in the cytoplasm and are mainly exported by Exportin 4 or Exportin 2 [21,27,28]. In addition, UAP56 and URH49 have been demonstrated to regulate the exportation of long and short circRNAs, respectively [29]. In contrast to linear RNAs, circRNAs are more resistant to RNA exonucleases, making them more suitable to be used as biomarkers or therapeutic targets [2,11,30,31]. Accumulating evidences have demonstrated that circRNAs play essential roles in a variety of biological processes via acting as miRNA sponges, transcriptional regulation, modulating alternative splicing, interacting with RBPs, and even encoding peptides [2,[32], [33], [34], [35], [36], [37], [38]].

Compared to mRNAs, ncRNAs, particularly circRNAs, are overall evolutionarily nonconserved [39]. However, with the development of high-throughput sequencing and related experimental techniques, more and more conserved lncRNAs (e.g., *XIST*, *MALAT1*, *NEAT1*) and circRNAs (e.g., *CDR1as/ciRS-7*, *circLARP1B*) have been identified [37,38,[40], [41], [42], [43], [44], [45]]. They always exhibit a high degree of sequence (or position) conservation, including the backsplice junction sequence of circRNAs, and perform analogous functions by similar mechanisms across species [37,38,[40], [41], [42], [43], [44], [45]]. Due to the limitations of research in human, conserved lncRNAs and circRNAs could be investigated in animal models to further elucidate underlying mechanisms, functional roles, and clinical applications. In this review, we focus on the physiological functions and mechanisms of conserved lncRNAs and circRNAs.

## 2 Mechanisms and functions of conserved lncRNAs

### 2.1 Transcriptional regulation

**Fig. 1:**
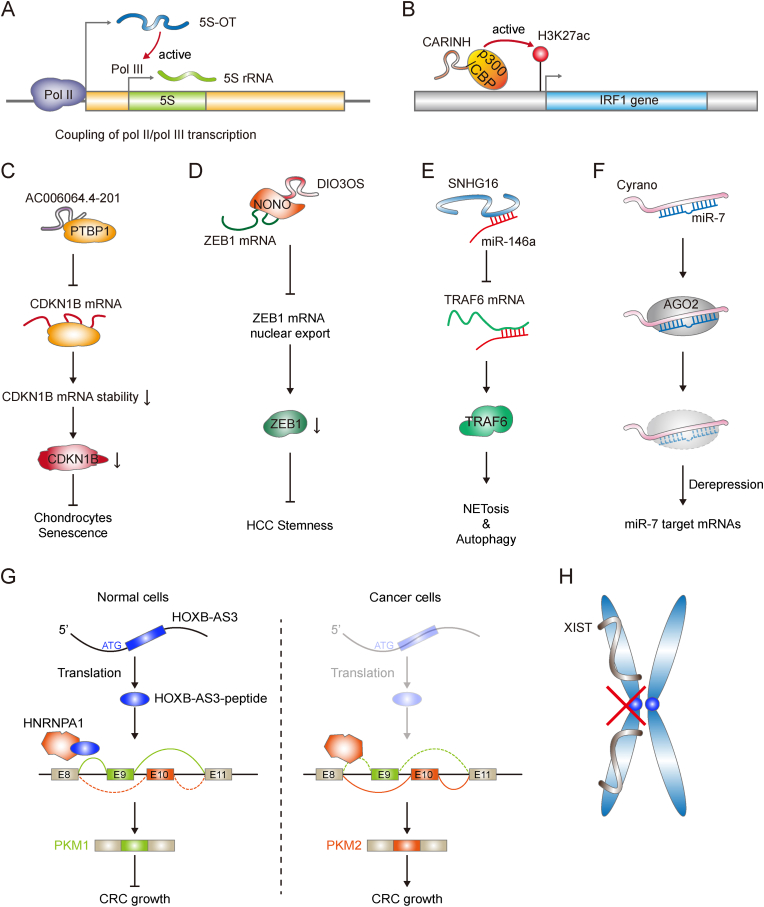
Alternative mechanisms of conserved lncRNAs. (A) The *cis* regulatory role of *5S-OT* in *5S* rRNA transcription. *5S-OT* is transcribed by pol II and *5S* rRNA is transcribed by pol III. (B) The lncRNA *CARINH* recruits p300/CBP to increase the deposition of H3K27ac at the IRF1 promoter, leading to the upregulated transcription of the IRF1 gene. (C) The lncRNA *AC006064.4-201* disturbs PTBP1 from binding to *CDKN1B* mRNA, resulting in the destabilization of *CDKN1B* mRNA and the downregulation of CDKN1B protein to inhibit chondrocyte senescence. (D) *DIO3OS* interacts with NONO to block NONO-mediated *ZEB1* mRNA nuclear export, thereby inhibiting HCC stemness. (E) *SNHG16* acts as a *miR-146a* sponge to relieve the repressive effect on its target TRAF6, ultimately regulating the NETosis and autophagy. (F) *Cyrano* promotes mature *miR-7* degradation through target RNA-directed miRNA degradation (TDMD) to de-repress the *miR-7* targeted mRNAs. (G) The putative lncRNA *HOXB-AS3* encoded polypeptide binds to HNRNPA1 and regulates the HNRNPA1-mediated splicing-regulation of PKM to inhibit colon cancer (CRC) growth. (H) *XIST* encapsulates the X chromosome to contribute to X chromosome inactivation.

Numerous lncRNAs exhibit the *cis* regulatory roles in gene transcription [11,16]. *5S* rRNA is an ancient non-coding RNA expressed in all domains of life, which is transcribed by RNA pol III from the corresponding internal promoter [46,47]. Hu and colleagues identified a transcript from *5S* rDNA loci named *5S-OT* (*5S* rRNA Overlapped Transcript) with extensive expressions from fission yeast to mammals [48]. *5S-OT* is a highly conserved lncRNA synthesized by RNA pol II and localized to the nucleus in human and mice [48]. Antisense oligonucleotides (ASO)-mediated *5S-OT* knockdown reduces the nascent *5S* rRNA level and human/murine *5S-OT* both co-localize to the *5S* rDNA gene cluster, indicating the existence of the RNA-mediated pol II-pol III coupling mechanism in gene transcription (Fig. 1A) [48]. Additionally, *5S-OT* also evolves *trans* regulatory roles in the anthropoidea suborder of primates due to the insertion of an antisense *Alu* element. Human *5S-OT* interacts with the splicing factor U2AF65 to modulate alternative splicing of hundreds of genes via anti-Alu/Alu pairing [48]. Based on this mechanism, the artificial RNA with a polypyrimidine tract (Py) site at the 5′ end and a gene-specific antisense sequence at the 3’ end could be used as an effective biotechnological tool to manipulate alternative splicing for several genes [48,49]. *FENDRR* (Fetal-lethal Noncoding Developmental Regulatory RNA) is another conserved lncRNA in human and mice [50,51]. The expression of *FENDRR* is decreased in the fibrotic lungs of patients with idiopathic pulmonary fibrosis and in an induced pulmonary fibrosis mouse model [50]. Depletion of *FENDRR* increases cellular senescence in human lung fibroblasts, while overexpression of human *FENDRR* reduces lung fibrosis in the pulmonary fibrosis mouse model [50]. Further investigation demonstrates that *FENDRR* decreases pulmonary fibrosis by competing with the binding of IRP1 to iron metabolism genes and then controlling the iron concentrations [50]. Additionally, murine *Fendrr* has also been found to directly form a RNA:dsDNA triplex at promoters of targeted genes associated with lung fibrosis [51]. Meanwhile, *Fendrr* and the Wnt-signaling pathway regulate these genes to participate lung fibrosis in a synergistic manner [51]. However, whether human *FENDRR* is involved in lung fibrosis via the RNA:dsDNA triplex requires further investigation.

Several lncRNAs such as long intergenic RNAs (lincRNAs) have *trans* regulatory roles [41,[52], [53], [54]]. For example, the *lincRNA-p21* is identified as one of the p53-regulated RNAs in response to DNA damage in mice [[52], [53], [54]]. The human orthologous *lincRNA-p21* is widely expressed in human fibroblasts and is induced upon DNA damage [54]. 930 overlapped genes are dysregulated upon RNA interference (RNAi)-mediated p53 knockdown and *lincRNA-p21* knockdown in mouse embryonic fibroblasts (MEFs) [54]. Further investigations reveal that *lincRNA-p21* suppresses numerous genes in the p53-dependent transcriptional response and induces cell apoptosis [54]. Mechanistically, *lincRNA-p21* interacts with HNRNPK, a p53 pathway repressor complex component, to modulate its genomic localization, thereby transcriptionally repressing p53-regulated genes [54]. *MALAT1* (Metastasis Associated Lung Adenocarcinoma Transcript 1) is another broadly recognized lincRNA, which is highly expressed in hepatic fibrosis tissues and interacts with DNMT1 both in human and mice [41]. Employing the hepatic fibrosis mouse model, Gong et al. have demonstrated that *MALAT1* recruits DNMT1 to the Glis2 promoter to induce DNA methylation, thereby downregulating Glis2 by repressing Glis2 transcription *in trans* and activating hepatic stellate cells to aggravate hepatic fibrosis [41].

### 2.2 Chromatin remodeling

LncRNAs have been reported to exert their functions by chromatin remodeling [11,16,[55], [56], [57], [58]]. For instance, the lncRNA *EPR* (Epithelial cell Program Regulator) is prominently expressed in epithelial tissues such as the mammary gland, gastrointestinal tract, lung, and kidney [57,58]. In both human and mice, the *EPR* level is significantly decreased in breast cancer cell lines compared to normal mammary gland cells and *EPR* overexpression reduces breast cancer cell proliferation [57]. Murine *Epr* recruits Smad3 to the promoter of Cdkn1a to enhance its transcription. Meanwhile, human *EPR* overexpression also induces Cdkn1a levels in murine cells [57]. When examined at the organismal level, the *Epr* genetic knockout mice (Epr^−/−^) demonstrate higher susceptibility to colitis and tumor formation under dextran sodium sulfate induction through tridimensional chromatin remodeling to decrease the expression of genes involved in mucus metabolism [58].

*CARINH* (Colitis Associated IRF1 antisense Regulator of Intestinal Homeostasis) is another mammal-conserved lncRNA, which is localized close to the 3’ end of the coding gene IRF1 in human and mice [59]. Both human and murine *CARINH* are highly expressed in lymphoid and mucosal tissues. Murine *Carinh* is upregulated in myeloid cells sorted from colitis colons than healthy ones, and human *CARINH* levels are increased in gut specimens from inflammatory bowel disease (IBD) patients than control biopsy tissues [59]. Moreover, *CARINH* RNA levels are positively correlated with IRF1 levels both in mouse myeloid cells and human monocyte THP-1 cells [59]. Mechanistic studies demonstrate that *Carinh* interacts with p300/CBP at the Irf1 locus to promote Irf1 expression by increasing H3K27ac deposition (Fig. 1B) [59]. Further investigation shows that IRF1 also directly binds to the promoter of *Carinh* and promotes its transcription. All these data indicate that *CARINH* and IRF1 form a feedforward loop, which is essential for against IBD both in human and mice.

### 2.3 mRNA stability

One mechanism of lncRNAs is to regulate mRNA stability [8,60,61]. The lncRNA *THOR* (Testis-associated Highly-conserved Oncogenic long non-coding RNA) is highly conserved in human, mice and zebrafish [60]. *THOR* is extensively expressed in the testis and a variety of human cancers, including non-small-cell lung cancer (NSCLC) and melanoma [60]. *THOR* knockdown results in a significant reduction of cell proliferation in NSCLC and melanoma cell lines. Further murine tumor xenografts derived from *THOR* knockout and overexpression cells support the proliferation-promoting activity of human *THOR* [60]. Moreover, transgenic *THOR* knockout in zebrafish leads to fertilization failure and confers resistance to melanoma development, whereas ectopic expression of human *THOR* accelerates melanoma development in zebrafish [60]. Mechanistically, *THOR* interacts with the RBP IGF2BP1 in both human and zebrafish to increase the stability of IGF2BP1's targets. Overall, *THOR* is a cancer/testis lncRNA and has undergone favorable evolutionary selection. The lncRNA *AC006064.4-201* (human) or *Gm49317-201* (mouse) is another conserved lncRNA with a conserved mode of action in mammals. Human *AC006064.4-201* is decreased in senescent and degenerated human chondrocytes and protects against Osteoarthritis [61]. Additionally, *Gm49317-201* knockdown results in an increase in the number of senescent mouse chondrocytes [61]. The lncRNA directly interacts with PTBP1 to perturb PTBP1 binding to *CDKN1B* mRNA, leading to a decrease of *CDKN1B* mRNA stability and protein level, and this regulatory axis is conserved between human and mice (Fig. 1C) [61].

LncRNAs could also regulate mRNA stability via RNA-RNA interaction [[62], [63], [64]]. Taking an example of the lncRNA *HAS2-AS1* (Hyaluronan Synthase 2 Antisense 1), human *HAS2-AS1* is downregulated in sunitinib-treated induced pluripotent stem cell-derived endothelial cells (iPSC-ECs), and the murine analogue *Has2os* is decreased in the sunitinib-induced vascular dysfunction mouse model [64]. Mechanistic studies have revealed that *HAS2-AS1* forms an RNA duplex with *HAS2* mRNA to increase *HAS2* mRNA stability through protection against ribonuclease degradation [64]. This study indicates that the *HAS2-AS1*/*HAS2* axis plays pivotal roles in mediating sunitinib-induced vascular toxicity [64].

### 2.4 mRNA exportation

LncRNAs are involved in mRNA nuclear exportation as well [65,66]. The conserved lncRNA *DIO3OS* (DIO3 Antisense RNA) is frequently downregulated in various cancers, including hepatocellular carcinoma (HCC) [66]. Low *DIO3OS* expression correlates with poor clinical outcomes in HCC [66]. Gain- or loss-of function studies reveal that *DIO3OS* significantly inhibits tumor development via its suppressive role in cancer stem cells (CSCs) by decreasing ZEB1 protein levels [66]. Mechanistically, *DIO3OS* binds to the NONO protein and reduces the *ZEB1* mRNA nuclear export mediated by NONO (Fig. 1D) [66]. The hydrodynamic tail vein injection (HTVI) HCC mouse model demonstrates that murine *Dio3os* also exerts a suppressive role in HCC tumorigenesis via the conserved *Dio3os*-Zeb1 axis [66].

### 2.5 Regulate miRNA activities

The regulatory effects of lncRNAs on miRNAs are mainly achieved by acting as miRNA sponges, acting as miRNA precursors, and mediating the degradation of miRNAs through target RNA-directed miRNA degradation (TDMD) [8,9,16,44,[67], [68], [69], [70], [71], [72]]. The conserved lncRNA *SNHG16* (Small Nucleolar RNA Host Gene 16) participates in the systemic lupus erythematosus-associated alveolar hemorrhage (SLE-AH) pathogenesis via regulating autophagy and neutrophil extracellular traps (NETs) formation [68]. Both human and murine *SNHG16* are upregulated in SLE-AH lung tissues with increased autophagy, apoptosis, and NETs formation [68]. Mechanistically, *SNHG16* serves as a *miR-146a* sponge to increase the levels of TRAF6, which is a potent inducer of NETosis (a program for NETs formation) and autophagy (Fig. 1E) [68]. A similar ceRNA mechanism also applies to another mammal-conserved lncRNA, *CARMA* (Cardiomyocyte Maturation-Associated lncRNA) [69]. Both human and murine *CARMA* contain conserved *miR-133a2* binding sites to decrease the expression of its neighboring gene, MIR-1-1HG, the host gene of *miR-133a2 in cis.* The *CARMA*/*MIR-133-a2* feedback loop contributes to the coordinated regulation of cardiogenic differentiation in ESCs [69].

The lncRNA *Cyrano*, also known as *OIP5-AS1* (OIP5 Antisense RNA 1), is broadly conserved in vertebrates [44,56,70]. It is expressed throughout the developing nervous system and is highly expressed in the brain [44,56,70]. Kleaveland and colleagues have utilized a series of genetic knockout mice to demonstrate that *Cyrano* triggers much more effective *miR-7* destruction via an extensively miR-7-paired site. By destabilizing *miR-7* levels through TDMD, *Cyrano* prevents inhibition of miR-7-targeted mRNAs and promotes the accumulation of *Cdr1as*, a circular RNA known to regulate neuronal activity (Fig. 1F) [44]. In addition, human *H19* is an imprinted lncRNA originating from the H19-IGF2 imprinting cluster of the genome [71,72]. In addition to functioning as a spliced full-length transcript, *H19* could also be a *miR-675* precursor for *miR-675* generation. A full predicted *H19* genomic sequence is also identified in wallabies, with approximately 51 % similarity to the human *H19*. Furthermore, the *H19* ortholog in therian species demonstrates preservation of both miRNA production and exon-intron structure, indicating functional selection for these characteristics [72]. Overall, lncRNAs associate with miRNAs to form complex regulatory networks in physiology and pathology.

### 2.6 Encode polypeptide

Although the vast majority of lncRNAs are thought to be non-coding, a small portion of lncRNAs exhibit the translational effects to encode polypeptides under certain circumstances [30,[73], [74], [75], [76], [77], [78]]. For example, the putative lncRNA *HOXB-AS3* (HOXB Cluster Antisense RNA 3) encodes a conserved and functional 53-aa peptide in primates [78]. The low HOXB-AS3-peptide level is correlated with a poor prognosis in CRC patients, and HOXB-AS3-peptide represses colorectal cancer (CRC) growth *in vitro* and *in vivo*. Mechanistic studies have revealed that the HOXB-AS3-peptide interacts with the hnRNPA1 protein and sequesters hnRNPA1 to modulate alternative splicing of pyruvate kinase M (PKM), resulting in the decreased PKM2 isoform inhibiting CRC growth (Fig. 1G) [78].

### 2.7 X chromosome inactivation

*XIST* (X-inactive Specific Transcript) and *TSIX* (Xist antisense RNA) are two well-characterized lncRNAs associated with X chromosome inactivation. They are transcribed from the same gene loci with distinct orientations. *XIST* is a sense transcript, and *TSIX* is an antisense transcript [40,79]. *XIST* contains conserved repeats and predominantly resides in the nucleus [40]. *XIST* gradually encapsulates X chromosome starting from the X inactivation center (XIC) followed by instant gene silencing (Fig. 1H) [40]. *TSIX* is conserved in the human XIC and mouse XIC [40]. *TSIX* prevents the accumulation of *XIST* on the future active female X chromosome through RNA-RNA interaction to maintain the active euchromatin state of the selected chromosome [79].

### 2.8 Other mechanisms

LncRNAs could coordinate with key regulators to perform various biological functions [8,11]. *NEAT1* (Nuclear Enriched Abundant Transcript 1) is a well-known conserved lncRNA that plays an important role in the development of several cancers, including lung cancer, breast cancer, and prostate cancer [42]. The accelerated glycolysis (also named Warburg effect) is one of the main metabolic changes observed in cancer [42]. Using human cell lines and mouse models, Park et al. found that *NEAT1* interacts with PGK1, PGAM1, and ENO1 proteins to facilitate the assembly of the PGK1/PGAM1/ENO1 glycolytic complex, resulting in a higher flux through the penultimate step in glycolysis [42]. Human *HOXA11AS* (Homeobox A11 Antisense) or murine *HOXA11os* is a conserved lncRNA transcript from the opposite strand of the HOXA11 gene locus in mammals [80]. In clinical samples, *HOXA11AS* is highly expressed in colon biopsies specimens and exhibits a significant reduction in those with ulcerative colitis [80]. Similarly, murine *HOXA11os* is highly expressed in the healthy colon, but downregulated to undetectable levels in colitis [80]. Under normal conditions, *HOXA11os* localizes to mitochondria and interacts with the complex I subunit, NDUFV1, to sustain its function [80]. *HOXA11os* deficiency mice demonstrate complex I deficiency, oxidative phosphorylation dysfunction, and mitochondrial reactive oxygen species production in colonic myeloid cells [80]. The conserved lncRNAs with functional roles and molecular mechanisms are summarized in Table 1.

**Table 1:** Conserved lncRNAs and circRNAs.

LncRNA	Species	Physiological or pathological functions	Molecular mechanism	Conservation	Refs
*5S-OT*	Eukaryote	Reduces the nascent *5S* rRNA level	Co-localizes to the *5S* rDNA cluster to modulate its transcription	Sequence, function, mechanism	[48]
	Primate	Associates with THP-1 cell differentiation	Interacts with U2AF65 to modulate alternative splicing via Alu/*anti*-Alu pairing		
*FENDRR*	Human, mouse	Decreases lung pulmonary fibrosis	Competes with IRP1 protein from iron metabolism genes	Position, function	[50]
*LincRNA-p21*	Human, mouse	Induces cell apoptosis	Interacts with HNRNPK to transcriptionally repress p53-regulated genes	Sequence, function, mechanism	[54]
*MALAT1*	Human, mouse	Activates hepatic stellate cells to aggravate hepatic fibrosis	Recruits DNMT1 to the Glis2 promoter to inhibit Glis2 transcription	Sequence, function, mechanism	[41]
*EPR*	Human, mouse	Reduces breast cancer cell proliferation	Recruits Smad3 to the Cdkn1a promoter to enhance its transcription	Sequence, function,	[57]
	Mouse	Inhibits susceptibility colitis and tumor formation	Remodels 3D chromatin to decrease expressions of some mucus metabolism genes	/	[58]
*CARINH*	Human, mouse	Against inflammatory bowel diseases	Interacts with p300/CBP at the IRF1 locus to promote IRF1 expression	Sequence, function, mechanism	[59]
*THOR*	Human, mouse, zebrafish	Promotes proliferation of non-small-cell lung cancer and melanoma cell lines, and contributes to fertilization and melanoma development in zebrafish	Interacts with IGF2BP1 to increase the stability of IGF2BP1's targets	Sequence, expression pattern, function, mechanism	[60]
*AC006064.4-201*	Human, mouse	Protects against Osteoarthritis	Perturbs PTBP1 binding to *CDKN1B* mRNA to decrease *CDKN1B* mRNA stability	Sequence, function, mechanism	[61]
*HAS2-AS1*	Human, mouse	Plays pivotal roles in mediating sunitinib-induced vascular toxicity	Forms an RNA duplex with *HAS2* mRNA to increase its stability	Position, function, mechanism	[64]
*DIO3OS*	Human, mouse	Inhibits tumor development via its suppressive role in cancer stem cells	Binds to the NONO protein and restricts the *ZEB1* mRNA nuclear export	Sequence, function, mechanism	[66]
*SNHG16*	Human, mouse	Increases autophagy, apoptosis, and NETs formation of systemic lupus erythematosus-associated alveolar hemorrhage lung tissues	Serves as a *miR-146a* sponge to increase the TRAF6 levels	Sequence, function, mechanism	[68]
*CARMA*	Human, mouse	Contributes to the coordinated regulation of cardiogenic differentiation in ESCs	Sponges *miR-133a2* to decrease the expression of its neighboring gene, MIR-1-1HG	Position, function, mechanism	[69]
*Cyrano*	Vertebrate	Regulates neuronal activity	Reduces *miR-7* levels by TDMD	Position, function, mechanism	[44,70]
*H19*	Human, wallaby, therian	Either acts as a growth suppressor or as a growth enhancer of tumor	Acts as a spliced full-length transcript or the *miR-675* precursor	Sequence, function, mechanism	[72]
*HOXB-AS3*	Primate	Inhibits colon cancer growth	Encodes a peptide to interact with HNRNPA1 to modulate alternative splicing of PKM	Sequence, function, mechanism	[78]
*XIST*	Human, mouse	Inactivates X chromosome	Encapsulates X chromosome	Function, mechanism	[40]
*TSIX*	Human, mouse	Activates euchromatin state of the selected chromosome	Prevents the accumulation of *XIST* through RNA-RNA interaction	Function, mechanism	[79]
*NEAT1*	Human, mouse	Facilitates glycolysis	Acts as a scaffold to promote PGK1/PGAM1/ENO1 glycolytic complex assembly	Sequence, function, mechanism	[42]
*HOXA11AS*	Human, mouse	Inhibits colitis	Localizes to mitochondria and interacts with the complex I subunit, NDUFV1, to sustain its function	Sequence, function, mechanism	[80]
*CARMN*	Human, mouse	Regulates the visceral smooth muscle cell (SMC) phenotype and contractility of the gastrointestinal tract	Interacts with MYOCD to regulate the SRF/MYOCD complex to facilitate the expression of SMC genes	Sequence, expression pattern, function, mechanism	[106]
*LNCGM1082*	Human, mouse	Is resistant to bacterial infection	Binds to NLRC4 and PKCδ to promote their interactions	Sequence, function, mechanism	[107]
*NUDT6*	Vertebrate	Regulates cell motility and differentiation in SMCs	Interacts with the CSRP1 protein and downregulates its levels	Sequence, function, mechanism	[108]
*CDR1as*	Mammal	Dampens neuronal activity	Sponges *miR-7* and is cleaved by *miR-671*	Sequence, function, mechanism	[43,44]
*circMEF2A1*	Mammal, chicken	Facilitates myogenesis	Modulates the miR-30a-3p/PPP3CA/NFATC1 axis	Sequence, function, mechanism	[81]
*circMEF2A2*		Promotes myogenic differentiation	Modulates the miR-148a-5p/SLIT3/ROBO2/β-Catenin axis		
*circBoule*	Mammal, fly	Regulates male fertility in flies and mice, and is downregulated in sperm from asthenozoospermic patients	Interacts with HSPs to facilitate their ubiquitination	Parental gene, function, mechanism	[87]
*circLARP1B*	Mammal	Facilitates metastasis and lipid accumulation of HCC	Destabilizes LKB1 mRNA via perturbing HNRNPD to modulate the AMPK-ACC signaling	Sequence, function, mechanism	[45]
*cia-cGAS*	Human, mouse	Safeguards dormant LT-HSCs from cGAS-mediated depletion	Interacts with DNA sensor cGAS to block its synthase activity	Sequence, function, mechanism	[88]
*circIPO11*	Human, mouse	Inhibits the self-renewal of liver CSCs and suppresses tumor propagation	Recruits TOP1 to GLI1 promoter to initiate GLI1 transcription	Sequence, function, mechanism	[89]
*circSLC45A4*	Human, mouse	Keeps neural cells in a progenitor state	Unknown	Sequence, function	[90]
*circARHGAP5-2*	Human, mouse	Maintains the global adipocyte gene program and facilitates lipid accumulation	Unknown	Sequence, function	[91]

## 3 Mechanisms and functions of conserved circRNAs

### 3.1 miRNA sponge

One mechanism of circRNAs is to act as miRNA sponges to de-repress miRNA targets [2,[18], [19], [20], [21]]. *CircMEF2A1* and *circMEF2A2* are backspliced from the *MEF2A* gene, are both evolutionarily conserved in human, macaque, mouse, rat, pig, and chicken [81]. *CircMEF2A1* serves as a ceRNA for *miR-30a-3p* to activate PPP3CA/NFATC1 signaling to facilitate myogenesis; *circMEF2A2* promotes myogenic differentiation via the miR-148a-5p-SLIT3/ROBO2/β-Catenin axis [81]. *CircMEF2A1* and *circMEF2A2* also regulate linear *MEF2A* expression by targeting *miR-30a-3p* and *miR-148a-5p*, respectively [81].

**Fig. 2:**
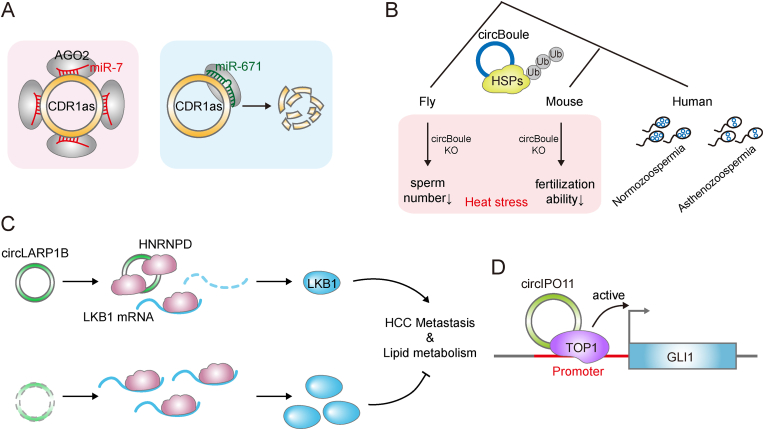
Molecular mechanisms of conserved circRNAs. (A) *CDR1as* functions as a *miR-7* sponge (Left). *MiR-671* associates with Ago 2 to mediate *CDR1as* degradation. (B) Conserved circBoule-HSP interaction and functional mechanisms form flies to mammals. *CircBoule* RNAs interact with HSPs to facilitate their ubiquitination. Genetic *circBoule* knockout flies and mice both demonstrate decreased male fertility, especially under heat-stress conditions. In addition, the *circBoule* level is significantly lower in sperm from patients with asthenozoospermia. (C) *CircLARP1B* binds to HNRNPD protein via two conserved motifs and inhibits HNRNPD binding to the 3′ UTR of *LKB1* mRNA. *CircLARP1B* destabilizes *LKB1* mRNA and downregulates LKB1 protein to promote hepatocellular carcinoma (HCC) metastasis and lipid accumulation. (D) *CircIPO11* increases GLI1 transcription by recruiting TOP1 to the GLI1 promoter.

The circRNA *CDR1as*/*ciRS-7* a well-characterized ceRNA, harbors more than 70 conserved binding sites of *miR-7* [37,38,43,44]. A number of studies have indicated that *CDR1as* acts as a miRNA sponge for *miR-7*, derepressing its targets and consequently impacting brain development [37,38]. In contrast, other studies have found that the knockout of *CDR1as* leads to decreased levels of *miR-7*, suggesting that *CDR1as* somehow protects *miR-7* from degradation [43,44]. In addition to numerous conserved *miR-7* sites, *CDR1as* contains a solitary conserved *miR-671* binding site to facilitate AGO2-mediated cleavage of the circRNA through almost perfect complementarity to *miR-671* (Fig. 2A) [43,44]. Interestingly, *CDR1as*, *miR-7* and *miR-671* coordinate with the lncRNA *Cyrano* to form a regulatory network in regulating neuronal activity [44]. In addition to regulating neuronal activity, *CDR1as* has been proposed to have oncogenic properties because it is highly expressed in tumors and its target, *miR-7*, is known to be a tumor suppressor [[82], [83], [84]]. However, Kristensen and colleagues pointed out that, in colon cancer, *CDR1as* is not expressed in cancer cells but instead is only expressed in stromal cells, while *miR-7* is only expressed in cancer cells [85,86].

### 3.2 Modulating RBP functions

CircRNAs always function by interacting with RBPs [2,11,[19], [20], [21]]. For instance, *circBoule* RNAs are generated from a conserved reproductive gene *Boule*, and are highly conserved and specifically expressed in testes from flies to mammals [87]. They directly bind to non-canonical RBPs heat-shock proteins (HSPs; Hsc4 and Hsp60C in flies, and HSPA2 in mammals) through conserved RNA motifs and then facilitate the ubiquitination of these HSPs to decrease their protein levels [87]. Genetic *circBoule* knockout flies and mice both lead to decreased male fertility, especially under heat-stress conditions. In addition, the *circBoule* level is significantly lower in sperm from patients with asthenozoospermia, and is negatively associated with the HSPA2 protein level in normozoospermic sperm (Fig. 2B) [87]. All these evidences reveal conserved molecular and physiological functions of *circBoule* RNAs in fertility protection, stress responses, and evolution.

Recently, Li and colleagues have identified a mammalian conserved circRNA named *circLARP1B*, which exhibits significantly higher levels in metastatic HCC [45]. *CircLARP1B* consists of exons 2–4 from the *LARP1B* gene and predominately localizes to the cytoplasm. HCC patients with higher *circLARP1B* levels had shorter five-year overall survival and disease-free survival rates [45]. *CircLARP1B* facilitates HCC metastasis through remodeling the lipid metabolism in a human HCC cell line and an induced HCC model of genetic knockout mice. Mechanistically, *circLARP1B* interacts with cytoplasmic HNRNPD via two functional sites and destabilizes *LKB1* mRNA via perturbing HNRNPD to modulate AMPK-ACC signaling in human and mice (Fig. 2C) [45]. Furthermore, AAV8-mediated hepatocyte-directed knockdown of *circLARP1B* or Lkb1 in HCC mouse models also demonstrated critical roles of the hepatocytic *circLARP1B* regulatory pathway in HCC metastasis and lipid metabolism, indicating that *circLARP1B* might be a potential therapeutic target for HCC [45].

### 3.3 Transcriptional regulation

Multiple circRNAs could regulate gene transcription [[19], [20], [21]]. The circRNA *cia-cGAS* is highly expressed in long-term hematopoietic stem cells (LT-HSCs) among human, mice, and rats [88]. The *cia-cGAS*-deficient mice exhibit decreased numbers of dormant LT-HSCs [88]. Under basal conditions, *cia-cGAS* interacts with DNA sensor cGAS to block its synthase activity in the nucleus, thereby safeguarding dormant LT-HSCs from cGAS-mediated depletion [88]. In the absence of *cia-cGAS*, cGAS binds to its own genomic DNA and catalyzes the synthesis of cGAMP products, which are released into the cytoplasm and then trigger the production of type I interferons, leading to LT-HSCs exhaustion [88]. *CircIPO11* is another conserved circRNA in human and mice with significant expression in both liver CSCs and HCC tumor tissues [89]. Downregulation of human *circIPO11* inhibits the self-renewal of liver CSCs and suppresses tumor propagation *in vitro* and *in vivo*. Mechanistic study reveals that *circIPO11* recruits TOP1, relaxing supercoiled DNA during gene replication and transcription, to the GLI1 promoter, thereby initiating GLI1 transcription and activating Hedgehog signaling for self-renewal maintenance of liver CSCs (Fig. 2D) [89].

### 3.4 Other circRNAs

*CircSLC45A4* is a highly conserved circRNA in human neuroblastoma cells and mouse embryonic cortex [90]. *CircSLC45A4* knockdown in human SH-SY5Y cells causes spontaneous differentiation. Depletion of *circSlc45a4* significantly reduces the basal progenitor pool in the developing mouse cortex [90]. *CircARHGAP5-2* is conservatively expressed in human and mouse adipocytes, which maintains the global adipocyte gene program, and facilitates lipid accumulation [91]. Although *circSLC45A4* and *circARHGAP5-2* are two functionally conserved circRNAs, the mechanisms of their function require further investigation.

## 4 Conclusions and perspectives

Current active research in lncRNAs and circRNAs has brought us various interesting findings implying that lncRNAs and circRNAs are of great importance in human diseases [30,[92], [93], [94], [95]]. Several of them are evolutionarily conserved and have essential roles in physiology and pathology [1,8,30,31]. We have systematically described a series of conserved lncRNAs and circRNAs, and elucidated their underlying molecular mechanisms (Table 1 and Fig. 1, Fig. 2).

Compared to mRNAs and circRNAs, lncRNAs are less conserved across species; thus, conserved lncRNAs might be more dispensable due to their survival under the selection pressure of evolution [1,8,30]. For instance, the lncRNA *Epr* knockout mice have demonstrated a higher susceptibility to colitis and tumor formation when induced with dextran sodium sulfate [58]. *HOXA11os* knockout mice have defective mitochondria in colonic myeloid cells, leading to hypersusceptibility to colitis [80]. Transgenic *THOR* knockout in zebrafish results in fertilization failure and provides resistance to melanoma development [60]. Additionally, despite the seemingly normal appearance of Cyrano^−/−^ mice, knockdown of conserved *Cyrano* in zebrafish causes a neurodevelopmental phenotype [44,70]. The organismal phenotype of conserved lncRNAs highlights their importance across species.

It has been demonstrated that ceRNA interactions occur when miRNA and target levels approach equimolar [96]. Given that miRNAs are typically present in high copy numbers within cells, if a lncRNA or circRNA is functioning as a miRNA sponge, it should contain multiple binding sites for the target miRNA, such as *CDR1as* [37,38,43,44]. Nevertheless, the majority of lncRNAs and circRNAs that are believed to act as miRNA sponges contain only a single or a few target miRNA binding sites and are expressed exclusively in specific tissues [96]. If lncRNAs or circRNAs are capable of inducing TDMD, as exemplified by Cyrano's action on *miR-7*, the necessity for numerous binding sites and high abundance could be negated [44,97]. However, it is noteworthy that a relatively small number of lncRNAs or circRNAs are fully complementary to the entire sequence of the target miRNA [98]. Consequently, most physiological alterations in the expression of individual circRNAs or lncRNAs will not impede the activity of miRNAs.

Thus far, a lot of *in vitro* and *in vivo* methods have been developed to validate ceRNA hypotheses, such as bioinformatics analyses, luciferase reporter assays, and correlation analysis of circRNA-miRNA-miRNA target genes [96,98]. However, some limitations of these research methods have led to questions about the credibility of the conclusions. If a circRNA has a negative correlation with a miRNA and a positive correlation with its target mRNA, this circRNA is always considered to act as a miRNA sponge. Nevertheless, it has recently been demonstrated that this correlation may result from the co-expression or mutual exclusion of subpopulations [85,86]. Bulk tissues are typically composed of a heterogeneous mixture of cell types. For example, colon and prostate tumor tissues often contain uneven proportions of muscle cells, which are known to be rich in circRNAs [99]. Consequently, the discrepancy in muscle cell content between different samples may give rise to an artifactual bias in circRNA expression [85,86,98,99]. Similarly, lncRNA expression is also cell-specific, which means that the analysis of lncRNA/circRNA expression and function using large tissue samples, such as tumor and non-tumor adjacent specimens, may result in erroneous conclusions.

Given that the majority of circRNAs overlap with mRNAs and the functions of most circRNAs are unknown, there is actually some controversy surrounding the question of whether circRNAs are conserved [39]. Here, we have reviewed a number of circRNAs that are expressed in more than one species and have analogous biological functions. One of them, the *CDR1as*, which has no well-expressed linear equivalent, has conserved functions across multiple species with analogous mechanisms [37,38,43,44]. These studies about conserved circRNAs have highlighted the potential importance of circRNAs.

Almost all reported conserved circRNAs with conserved roles in multiple species are exonic circRNAs (EcircRNAs). However, up-to-date, no conserved circRNAs have been reported to produce conserved polypeptides, and we are looking forward to future reports on translationally competent conserved circRNAs. In addition to EcircRNAs, Exon-Intron circRNAs (EIciRNAs), circular intronic RNAs (ciRNAs), and mitochondrial-encoded circRNAs (mecciRNAs) are another three well-recognized subclasses of circRNAs [2,21]. EIciRNAs in the nucleus are reported to promote gene transcription *in cis* via interacting with U1 small nuclear ribonucleoprotein [2,35]. *CircRNF217*, an EIciRNA in the cytoplasm, was reported to play *trans* roles as a *miR-130-3p* sponge to facilitate antibacterial responses in teleost fish [100]. Interestingly, *circCAMSAP1* is conserved between human and mice, but only human *circCAMSAP1* shows intron retention [101], indicating the complex regulatory mechanism in intron retention of EIciRNAs. Although ciRNAs are widely expressed across species [2,102,103], conserved ciRNAs are rarely reported, largely owing to the lower conservation of intronic sequences compared to exonic sequences [102,103]. MecciRNAs are extensively expressed in human, mouse and zebrafish [22,104]. Even though the mitochondrial DNA sequence performs high similarity between human and mice, there are no conserved mecciRNAs with conserved functions reported [105].

In conclusion, the summarized functions and mechanisms of conserved lncRNAs and circRNAs somehow provide new insights into the RNA world from an evolutionary perspective.

## CRediT authorship contribution statement

**Jingxin Li:** Writing – original draft, Data curation, Conceptualization. **Xiaolin Wang:** Writing – review & editing, Project administration, Conceptualization.

## Declaration of competing interest

The authors declare that they have no known competing financial interests or personal relationships that could have appeared to influence the work reported in this paper.
